# Unraveling the genomic diversity and virulence of human fungal pathogens through pangenomics

**DOI:** 10.1371/journal.ppat.1012313

**Published:** 2024-07-11

**Authors:** Marion Perrier, Amelia E. Barber

**Affiliations:** 1 Junior Research Group Fungal Informatics, Institute of Microbiology, Friedrich Schiller University, Jena, Germany; 2 Cluster of Excellence Balance of the Microverse, Friedrich Schiller University, Jena, Germany; Vallabhbhai Patel Chest Institute, INDIA

## Introduction

Fungi are ubiquitous in all major biomes where they play beneficial roles, but they can also infect plants, insects, and animals. Notably, only a small fraction of the fungal kingdom causes life-threatening infections in humans. Human pathogenic fungi are a growing threat to humanity as advances in modern medicine increase the number of patients who are at risk for fungal infections. The mortality rate associated with these infections is generally high, mainly due to limited therapies and increasing antifungal resistance. Fungal pathogens, like most microbes, exhibit a wide genetic diversity and a correspondingly broad range of phenotypic differences among strains of the same species, including varying levels of virulence. The cumulative impact of these differences on the virulence of fungal pathogens remains poorly understood. However, a better understanding of the genomic differences that underlie virulence differences may ultimately lead to improved management of human fungal infections.

## What is a pangenome and why are they relevant to the study of human pathogenic fungi?

**Fig 1:**
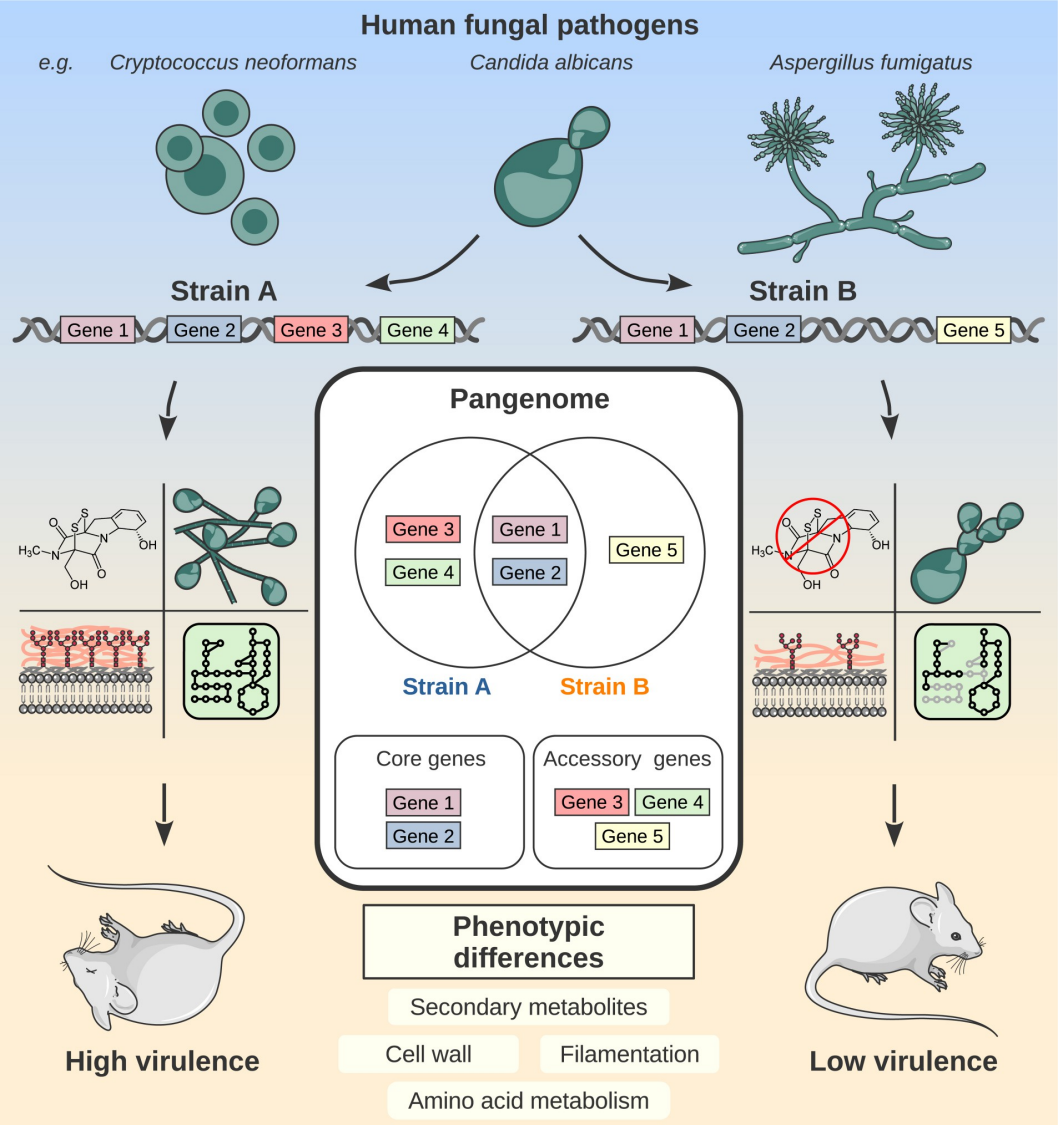
Pangenomic differences impact fungal virulence phenotypes. Illustrative example of the genomic differences between 2 strains of the same species and how they can lead to phenotypic differences in virulence. A schematic representation of a pangenome is shown in the middle. This figure uses original and modified drawings from Servier Medical Art, freely available under CC BY 4.0.

Pangenomes were first described in 2005 and have since been widely used to study prokaryotic microbes [1]. However, they have only recently been extended to eukaryotic organisms due to their larger genome size and complexity [2]. A pangenome is defined as the total collection of genes within a given phylogenetic group. It consists of a core genome, which are genes common to all strains, and an accessory genome, which are genes found only in a subset of strains (Fig 1). The core genome is mainly composed of essential genes and those involved in vital cell functions [3]. The function of accessory genes in eukaryotic organisms is not yet fully understood, but is hypothesized to be involved in fungal adaptation to the host or environment [4]. Accessory genes may also play roles in communication, pathogenicity, and antifungal resistance. The knowledge gap regarding the accessory genome in human pathogenic fungi prevents us from answering why some strains are more virulent than others and, more broadly, why strains of the same species exhibit different phenotypes (Fig 1). One way to answer these questions is to use pangenomes as a comparative method to analyze genomic differences between strains of the same species. Pangenomes can also serve as a reference for experimental studies that take diversity into account when investigating the roles of both conserved and variably present genes.

## Pangenomes highlight interstrain genomic diversity

**Fig 2:**
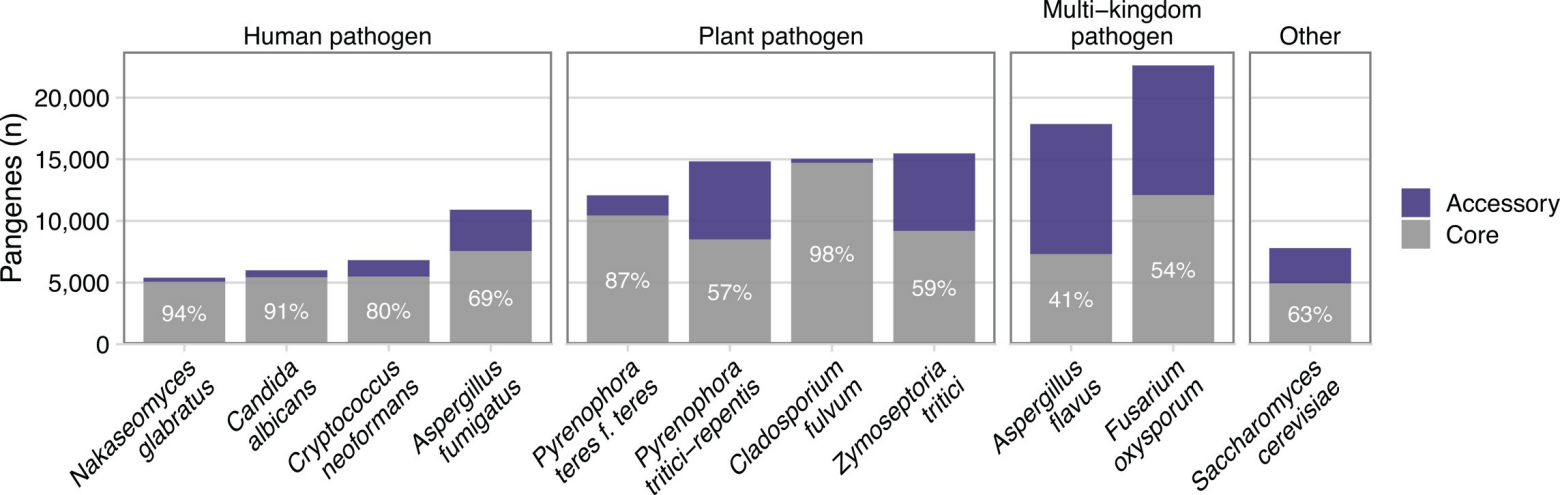
Variation in the pangenomes of human fungal pathogens. Bars show the total number of pangenes and their fill indicates the proportion of core and accessory genes. The core genome’s relative proportion is also indicated as a numerical value. Pangenome data for *N*. *glabratus* from [12] *C*. *albicans* and *C*. *neoformans var grubbii* from [3], *A*. *fumigatus* from [6], *P*. *teres f*. *teres* from [13], *P*. *tritici-repentis* from [14], *C*. *fulvum* from [11], *Z*. *tritici* from [15], *A*. *flavus* from [8], *F*. *oxysporum* from [10], and *S cerevisiae* from [16].

Fungi show considerable genomic variation, even within the same species [5]. Since pangenomes have only recently been extended to eukaryotes, only a few human fungal pathogens have been studied, but they have revealed highly variable ratios of core and accessory genes. The commensal fungi *Nakaseomyces glabratus* (formerly *Candida glabrata*) and *Candida albicans* have lower fractions of accessory genes with 6% and 9% of their total pangenomes, respectively (Fig 2). For environmental human fungal pathogens, the observed fraction of accessory genes is higher. In *Cryptococcus neoformans var*. *grubii*, accessory genes make up 19% of the pangenome and they account for 28% to 31% of the *Aspergillus fumigatus* pangenome [3,6,7]. A similarly wide range in the fraction of accessory genes is observed in the pangenomes of plant fungal pathogens, from 13% for *Pyrenophora teres f*. *teres* to 41% in *Zymoseptoria tritici*. The multi-kingdom plant and human pathogen *Aspergillus flavus* has the highest proportion of accessory genes described for a fungal pangenome, making up 59% of the total gene content for the species [8]. However, variable taxonomic definitions affect pangenome size and the degree of gene conservation reported. For example, 54% of genes were described as core in the *Fusarium oxysporum* pangenome [9], but other researchers would have classified the genomes analyzed in this study as belonging to many, distinct species as part of the *F*. *oxysporum* species complex. Interestingly, *Z*. *tritici*, *Fusarium* spp., and other plant-pathogenic fungi encode part of their accessory genome on discrete chromosomes that show presence–absence variation [9–11]. In human fungal pathogens, the accessory genome has so far only been described throughout the genome, with an enrichment in subtelomeric regions [3,6].

## Pangenomes reduce bias introduced by single, linear reference genomes

Omics analysis of eukaryotic organisms typically relies on a linear reference genome, which is the genome from a single strain that has been previously assembled, annotated, and made publicly available. This approach reduces computational time and complexity by simply mapping the genomic or transcriptomic sequence to the reference. However, using a single genome as a reference has a major drawback: it introduces bias when analyzing sequencing data from genetically divergent, non-reference strains. Reads containing more sequence polymorphisms are mapped to the reference at a lower rate, leading to an underestimation of genetic divergence [17]. This bias is even more pronounced in species with high genomic diversity, such as *A*. *fumigatus*, where a large number of accessory genes are simply ignored because they are not in the reference genome. In light of this, recent work has focused on the development of reference pangenomes. These can be implemented simply as a collection of CDSs (i.e., coding sequences) or as graph-based pangenome structures, both of which can be used for the alignment of sequencing reads [18]. Regardless of the implementation, pangenomes encourage a move away from single reference strains and toward a better understanding of how genetic variation affects a strain’s phenotype, including differences in virulence or fitness under stress.

## Pangenomes can elucidate phenotypic variation within a species

For many human fungal pathogens, there is marked phenotypic heterogeneity among commonly used strains, including variation in virulence traits [5–7,19]. There is a lack of knowledge about the accessory genes of human fungal pathogens because most research is done with reference strains. In *A*. *fumigatus* and *A*. *flavus*, there is remarkable presence–absence variation in many functional categories, including secondary metabolism genes. In *A*. *fumigatus*, this variation includes the presence of the virulence-associated factor gliotoxin [6,20] and in *A*. *flavus* the carcinogenic aflatoxin [21]. The accessory genome of *N*. *glabratus* contains many adhesion proteins, which are important virulence factors, and includes 4 novel adhesion groups [12,22]. In *C*. *albicans*, the natural loss of *ERG1*, a key regulator of filamentous growth and virulence, transforms a pathogen into an avirulent commensal [19]. A final example of the contribution of accessory genes to fungal virulence is demonstrated by the plant pathogen *F. oxysporum*. The virulence factors responsible for the ability to infect tomato plants are conserved only in strains that cause tomato wilt and are located on a small, accessory chromosome. The accessory genes can even be transferred during co-incubation between a strain lacking them and one possessing them, transforming a non-pathogen into a pathogen of tomato [23].

## Pangenomics helps to understand the evolutionary history of human fungal pathogens

The origin and maintenance of fungal accessory genes remains an open question. In fungal pathogens, as in eukaryotes in general, the accessory genome is primarily driven by both clonal and sexual recombination, gene duplication, and transposons and the differential contributions of these processes likely influences the proportion of accessory genes in a species. In contrast to prokaryotes, horizontal gene transfer (HGT) plays a lesser role in eukaryotic pangenome evolution [3,4,24]. The accessory genome likely contributes to the ongoing evolutionary arms race between host and pathogen, as demonstrated by the pangenomic studies of plant pathogenic fungi [10,13]. Hosts, including humans, are constantly evolving new strategies to recognize and eliminate pathogenic microbes, while at the same time pathogenic microbes are evolving new mechanisms to cause disease. For human-associated pathogenic fungi such as *N*. *glabratus*, new accessory adhesion genes may emerge due to host selective pressures if they confer a fitness advantage. In contrast, human fungal pathogens that primarily live in the environment are more likely to have pangenomes driven by their environmental fitness, rather than human habitats. The environment of these organisms is highly variable and constantly changing, potentially promoting the evolution of larger pangenomes to cope. Studies of the insect-pathogenic *Metarhizium* genus support this, as generalist species, which can survive in many habitats, have larger accessory genomes than their specialist counterparts [25,26]. This higher proportion of accessory genes in environmental organisms is also supported by a comparative study of 126 bacterial species, in which lifestyle was the largest determinant of pangenome evolution, and free-living species had larger and more fluid pangenomes than host-associated species [27]. How adaptive and/or neutral processes interact to collectively exert environmental impacts on pangenomes is an open question and may vary among species.

## The path ahead: Achieving widespread implementation of pangenomics

The use of pangenomes opens exciting new perspectives for gaining important functional insights into human fungal pathogens. However, there are still challenges that need to be overcome to realize their full potential:

### Lack of functional annotation and experimental characterization of accessory genes

The study of accessory genes is crucial to understanding phenotypic heterogeneity in fungal pathogens. However, researchers have historically used a limited number of reference strains. Pangenomes will improve our understanding of accessory genes and facilitate experimental work using non-reference strains. However, testing multiple strain backgrounds still requires considerable labor. Hopefully, high-throughput experimental techniques like robotics can offset this in the future.

### Genomic data availability and quality vary

The number of genomes, their representativity within the species, and their quality all impact the accuracy of the pangenome. Demonstrating this, the percentage of accessory genes for *A*. *fumigatus* grew from 17% for 12 genomes [3] to 31% for 300 genomes [6]. The complex nature of fungal genomes is another challenge. The annotation of eukaryotic genomes is an intricate and multi-step process due to eukaryotic genome features such as repetitive elements, complex regulatory elements, and intron-exon gene structures. Poorly annotated or incomplete genomes may overestimate the fraction of accessory genes when core genes are missing or misannotated.

### Lack of methodological “gold standard” and curation of pangenomic data

The field of pangenomics is relatively new for eukaryotic organisms. Bioinformatic tools in the field are constantly evolving and expanding and there is no “gold standard.” The most common approach is to search for orthologous coding sequences, independent of their genomic location. Other tools use a syntenic approach, which reflects the origins of pangenomics in prokaryotic organisms where genes are organized into operons. Graph-based pangenomes have recently been introduced to encode the genomic variation into a single reference structure. Comparing findings across studies is difficult due to the lack of consensus in methodology. Furthermore, there is no database that curates pangenomic data for fungal pathogens. However, the *Saccharomyces* Genome Database recently implemented a method to incorporate accessory genes that are absent from the reference, providing a promising solution [28].

In conclusion, fungal pangenomes are a recent but rapidly expanding field with the potential to reveal novel insights into their evolution, pathogenesis, and phenotypic heterogeneity.
